# Duration of infertility and assisted reproductive outcomes in non-male factor infertility: can use of ICSI turn the tide?

**DOI:** 10.1186/s12905-022-02062-9

**Published:** 2022-11-28

**Authors:** Liting Zhang, He Cai, Wei Li, Li Tian, Juanzi Shi

**Affiliations:** The Assisted Reproduction Center, Northwest Women’s and Children’s Hospital, Affiliated With Xi’an Jiaotong University, Xi’an, China

**Keywords:** Duration of infertility, ICSI, Non-male factor, Fertilization, Live birth

## Abstract

**Background:**

Intracytoplasmic sperm injection (ICSI) is increasingly used among in vitro fertilization (IVF) cycles without male factor infertility. For couples with prolonged infertility duration, the preferred insemination method may vary across laboratories and clinics. We analyzed whether ICSI is effective for non-male factor infertility with long infertility duration.

**Methods:**

Seventeen thousand four hundred seventy-seven IVF/ICSI cycles from women with non-male factor infertility were included, of these 4177 women with infertility duration ≥ 5 years were in the final analysis. Primary outcome was the live birth rate after first embryo transfer. Secondary outcomes were rates of clinical pregnancy and fertilization.

**Results:**

A nonlinear relationship was observed between infertility duration and IVF fertilization rate, which decreased with infertility years up to the turning point (4.8 years). 4177 women with infertility ≥ 5 years were categorized by IVF (*n* = 3806) or ICSI (*n* = 371). Live birth rate after first embryo transfer was 43.02% in ICSI and 47.85% in IVF group (adjusted odds ratio (aOR), 0.91; 95% confidence interval (CI), 0.72–1.15). Fertilization rate per metaphaseII (aOR, 1.10; 95% CI, 0.86–1.40) and clinical pregnancy rate (aOR, 0.89; 95% CI, 0.71–1.13) were similar between the two groups. Sensitive analyses (women ≥ 35 years) did not show a benefit of ICSI over IVF.

**Conclusions:**

Women with infertility exceeding 4.8 years had decreased incidence of IVF fertilization. The use of ICSI showed no significant improvement in fertilization and live birth rates for non-male factor couples with ≥ 5 years of infertility.

**Supplementary Information:**

The online version contains supplementary material available at 10.1186/s12905-022-02062-9.

## Background

Infertility is defined as the inability to conceive within 1 year of unprotected intercourse [1]. That definition is based on the estimation that 85% of the pregnancies occur within the first year in the fertile period [2]. After that, 10–15% of the couples are defined as infertile but pregnancy rates among them will reach nearly 55% in the next 3 years. After 4 years, 5% of the couples are definitively infertile with nearly no chance of becoming spontaneously pregnant [3]. On this basis, the longer the interval, the lower is the probability of conception and a prolonged duration of infertility has also been proposed as an indication to perform assisted reproductive technology (ART) [4]. Duration of infertility, which related to the severity of infertility, however, has received less attention.

An early systematic review and meta-analysis found a negative association between duration of infertility and IVF pregnancy rate (odds ratio (OR): 0.99, 95% confidence interval (CI): 0.98–1.00), suggesting that extending the infertility duration decreases the pregnancy chances in IVF [5]. However, only two small studies were included and whether the prolonged interval is associated with reduced fertilization success in IVF was not evaluated. Reliable information on this issue is lacking.

With the rationale that intracytoplasmic sperm injection (ICSI) is likely to reduce the likelihood of poor fertilization and to comfort both the patient and the physician, many clinicians tend to use ICSI as the preferred method of fertilization in couples with prolonged duration of infertility [6–8]. ICSI was initially developed for male factor infertility and then there has been an increase in the use of ICSI for all causes of infertility [9–11].

T he Practice Committee of the American Society for Reproductive Medicine suggested that there is insufficient evidence to support the routine use of ICSI in patients without male factor infertility [12]. Tannus et al. [13] evaluated the role of ICSI for non-male factor infertility in women aged ≥ 40 years, and they failed to show an advantage of ICSI over IVF for the sole indication of advanced maternal age. However, there are some situations where ICSI may be beneficial. According to the ASRM, ICSI could be used for ‘selected female factors including, but not limited to, morphologic anomalies of the oocyte, and anomalies of the zona pellucida’[14]. They also stated that ICSI might be indicated in couples with poor fertilization in a prior IVF cycle without detectable abnormalities of semen parameters. The real challenge in practice is to identify which specific group of patients are likely to have poor fertilization when they are undergoing the first cycle of IVF/ICSI treatment.

Currently there is no consensus on the threshold of infertility duration in ART treatment. The preferred insemination method in prolonged infertility duration may vary across laboratories and clinics. Therefore, this study aims to determine the duration of infertility and corresponding effects on fertilization rate in women with non-male factor undergoing ART. We hypothesized that long duration could decrease the rate of fertilization and if so, to explore whether ICSI could potentially improve fertilization rate and reproductive outcomes in couples with long duration of infertility.

## Materials and methods

### Study population

**Fig. 1:**
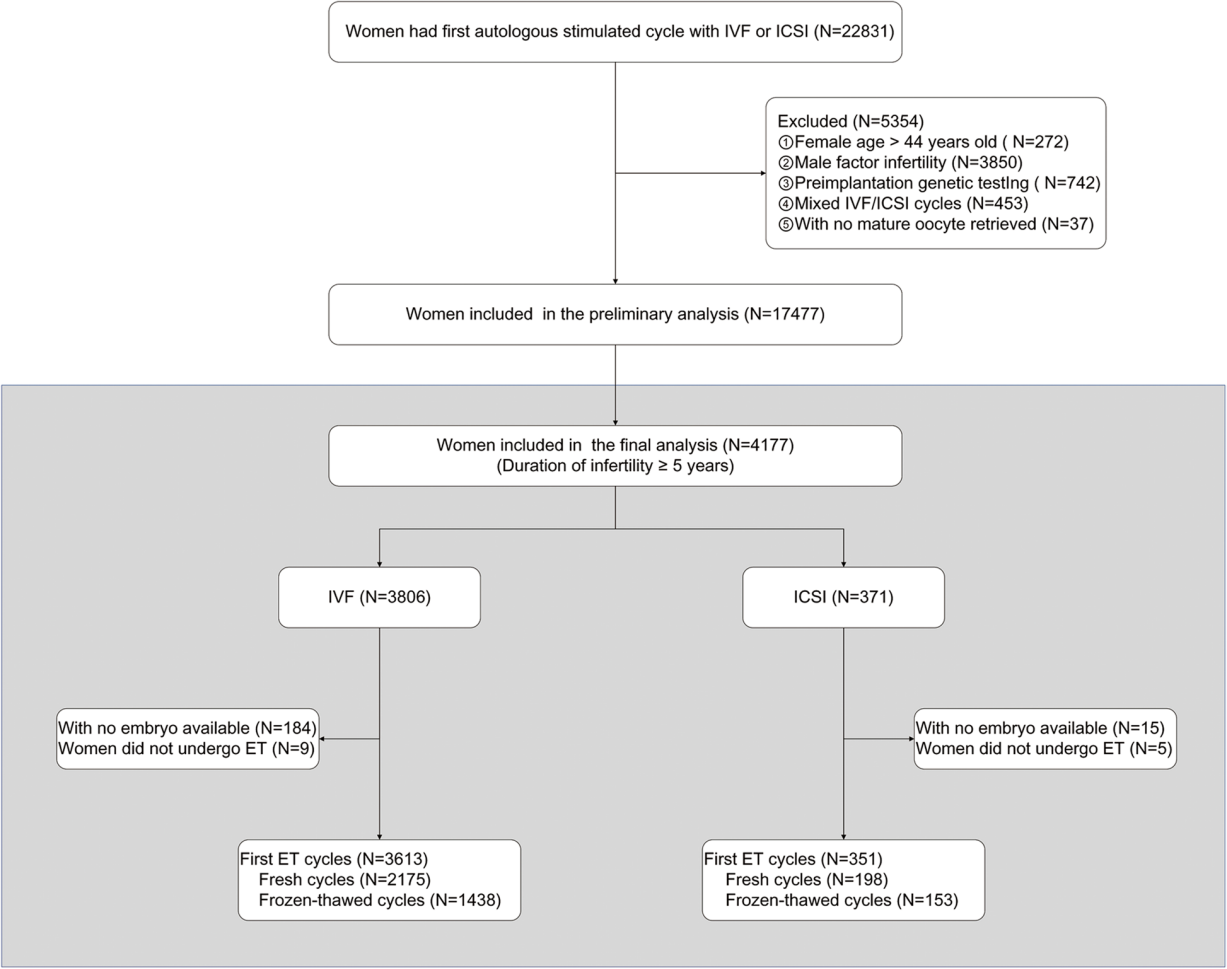
Patient flow through the study. Embryo transfer (ET)

A retrospective cohort study was conducted at the Northwest Women’s and Children’s Hospital. All women with non-male factor infertility who had ovarian stimulation with IVF or ICSI between January 2017 and December 2020 were evaluated for possible inclusion. The sperm analysis had met the criteria of the guidelines of WHO manual: total sperm count of at least 39 million, concentration ≥ 15 × 10^6^/ml, total motility ≥ 40%, progressive motility ≥ 32%, morphology ≥ 4% normal forms [15]. Women with non-male factor infertility having specific diagnoses including tubal factor, ovulatory disorder, diminished ovarian reserve, endometriosis, unexplained and others. Other inclusion criteria for the preliminary analysis were as follows: autologous cycles where couples used their own oocytes and sperm; first cycle of ovarian stimulation. Exclusion criteria included: women aged ≥ 42 years; mixed IVF-ICSI cycles (where oocytes from one oocyte retrieval were fertilized with split insemination (IVF/ICSI); preimplantation genetic test (PGT) cycles; cycles with frozen oocytes or sperm samples and women with no one mature oocyte retrieved (Fig. 1). Baseline characteristics, cycle characteristics, and pregnancy data were extracted from the electronic medical records.

### Stimulation protocol and embryology procedures

The ovarian stimulation protocol has been described previously [16]. Ovarian response was monitored by serial ultrasound examination and hormone measurement. 10,000 units of human chorionic gonadotrophin or 250 μg r-hCG was administered in patients when three follicles were > 18 mm. Oocyte retrieval was carried out 32–36 h following trigger. Women received either IVF or ICSI treatment depending on physician’s or patient’s preference. Cumulus stripping or insemination was performed 2 h after oocyte retrieval while fertilization was assessed after 16–18 h in the ICSI group and the conventional IVF group. Culture of zygotes was performed in drops of G1 medium (Vitrolife Ltd, Gothenburg, Sweden) till it was the third day. Embryos cultured beyond day 3 were transferred to blastocyst medium (sequential medium). The decision to culture for 3–5 days depended on developmental rate and morphological quality of the cohort. The embryo assessment methods have been described in detail elsewhere [17, 18]. The vitrification, warming procedure, endometrial preparation and embryos transfer procedures was done according to standard protocols [19].

### Variables and outcome measures

Duration of infertility was determined as the time from these couples’ first attempt to conceive to ovarian stimulation [20], and it was taken as a continuous measurement variable in the analysis. The primary outcome measure was live birth rate after the first embryo transfer (ET). Secondary outcomes were clinical pregnancy and fertilization rate (two pronuclei/MetaphaseIIoocyte). Clinical pregnancy was defined as gestational sac confirmed by ultrasound at 6 weeks gestation. Live birth was defined as any birth event in which at least one baby is born alive [21].

### Statistical analysis

We applied a two-piecewise linear regression model to examine the threshold effect of infertility duration on fertilization rate using a spline smoothing function. The turning point was determined with the use of trial and error, then choosing the turning point that provided the maximum model likelihood. Data are presented as mean ± SD or median (interquartile range) for continuous variables and as n (%) for categorical variables. The Student t test or Kruskal–Wallis rank test was used for parametric and nonparametric data, respectively. The Chisquare test or Fisher exact test for categorical variables was used for each group. Outcomes were compared with logistic regression analysis, controlling for confounding effects that included female and male age, infertility duration, gravidity, smoking, antral follicular count (AFC), the number of oocyte retrieved, number of MII oocytes, number and stage of embryos transferred. We also controlled for body mass index (BMI) due to its potential clinical significance although these were similar between the groups. Female age (years) (< 30, 30–34, 35–37, 38–40 and 41–44) and BMI (kg/m^2^) (< 18.5, 18.5–24.9, 25–29.9, 30–34.9 and ≥ 35) were also categorized for the clarity of data analysis. A sensitive analysis that restricted to women aged ≥ 35 years old was performed. Crude odds ratios (OR) and adjusted ORs (AOR) with 95% confidence interval (CI) were calculated. *P* value was considered significant if < 0.05.

Statistical analysis was performed using the Empower Stats (www.empowerstats.com, X&Y solutions, Inc. Boston MA) and R software version 3.6.1 (http://www.r-project.org).

## Results

### The impact of infertility duration on fertilization rate

**Fig. 2:**
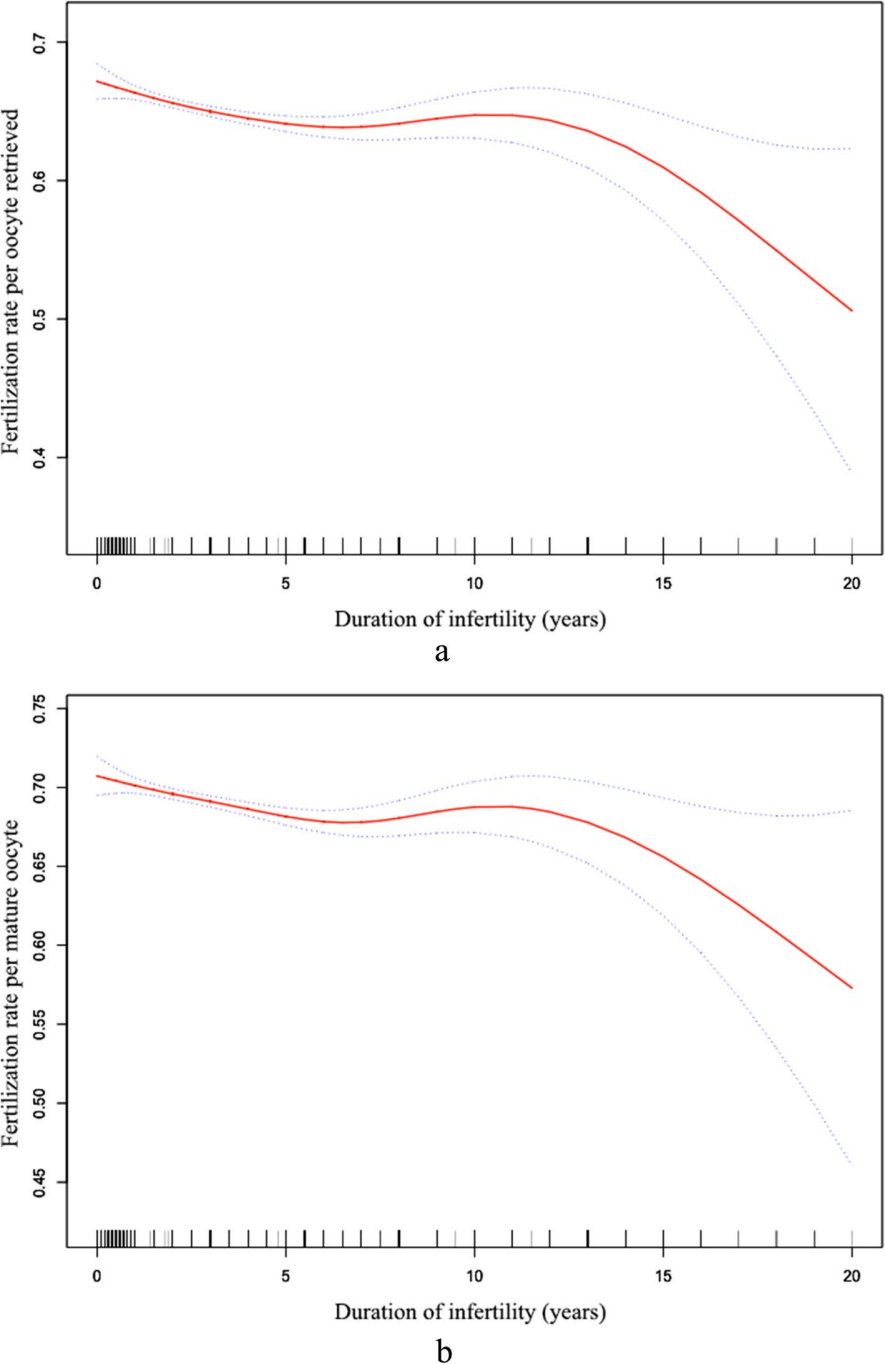
Associations between infertility duration and IVF fertilization in non-male factor infertility. **a** Infertility duration and 2PN rate per oocyte retrieved; **b** Infertility duration and 2PN rate per metaphaseII oocyte. The solid curve represents the adjusted fertilization rates, and the dashed curves represent the 95% confidence interval. The models are adjusted for female age, male age, number of oocytes retrieved and mature oocytes

During the study period, 17,477 non-male infertile patients had their first stimulated cycle were included in the preliminary analysis (16,406 undergoing IVF and 1071 undergoing ICSI). The median duration of infertility of women undergoing ICSI was 3 years (interquartile range, 2–5) and 3 years (interquartile range, 2–4) in women undergoing IVF. The nonlinear relationship between duration of infertility and fertilization rate (2PN/MIIoocyte and 2PN/oocyte retrieved) in IVF cycles was observed, the fertilization rate in IVF cycles decreased with years of infertility up to the turning point (4.8 years) adjusting for female and male age, numbers of oocytes retrieved and/or MIIoocytes (Fig. 2). The relationships between infertility duration and ICSI fertilization (2PN/MIIoocyte or 2PN/oocyte collected) were not significant. The regression coefficient after adjusted for potential covariates is shown in Additional file 1.

### ICSI versus IVF in women with infertility duration ≥ 5 years

#### Demographics and cycle characteristics

After selecting for patients with non-male factor infertility duration ≥ 5 years, we found 4177 women eligible (371 undergoing ICSI treatment and 3806 undergoing conventional IVF). In the ICSI group (371 started cycles), there were 198 fresh and 153 frozen transfers. In the IVF group (3806 started cycles), there were 2175 fresh and 1438 frozen transfers (Fig. 1). Baseline characteristics are presented in Table 1. ICSI treated women were relatively older (32.22 years vs. 31.74 years) and with longer duration of infertility (5–8 years vs. 5–7 years) than women treated with IVF.

**Table 1:** Demographics and cycle characteristics of patients with infertility ≥ 5 years

	IVF	ICSI	*P*-value
No. of patients	3806	371	
Years of treatment, n (%)			< 0.01
2017	863 (87.17)	127 (12.83)	
2018	988 (90.98)	98 (9.02)	
2019	1071 (92.01)	93 (7.99)	
2020	884 (94.34)	53 (5.66)	
Female age (years)	31.74 ± 3.82	32.22 ± 4.27	0.02
< 30, n (%)	1096 (28.80)	101 (27.22)	< 0.01
30–34	1873 (49.21)	159 (42.86)	
35–37	535 (14.06)	61 (16.44)	
38–40	219 (5.75)	37 (9.97)	
41–43	83 (2.18)	13 (3.50)	
Male age (years)	33.44 ± 4.49	34.58 ± 5.22	< 0.01
Body mass index (kg/m2)	23.36 ± 3.68	23.60 ± 3.49	0.22
< 18.5, n (%)	236 (6.20)	12 (3.23)	0.22
18.5–24.9	2463 (64.71)	246 (66.31)	
25–29.9	888 (23.33)	96 (25.88)	
30–34.9	187 (4.91)	14 (3.77)	
≥ 35	23 (0.60)	2 (0.54)	
Missing	9 (0.24)	1 (0.27)	
Education level, n (%)			0.63
Primary	183 (4.81)	22 (5.93)	
High school	2820 (74.09)	272 (73.32)	
College	803 (21.10)	77 (20.75)	
Smoker n (%) ^a^	862 (22.65)	67 (18.06)	0.04
Gravidity, n (%)			
0	2147 (56.41)	231 (62.26)	0.07
1	920 (24.17)	83 (22.37)	
≥ 2	739 (19.42)	57 (15.36)	
No. of prior live births, n (%)			0.02
0	3081 (80.95)	322 (86.79)	
1	660 (17.34)	44 (11.86)	
≥ 2	65 (1.71)	5 (1.35)	
AFC, n	13.21 ± 6.49	12.87 ± 6.39	0.35
Infertility duration^b^, years	6 (5–7)	6 (5–8)	< 0.01
Causing of infertility, n (%)			< 0.01
Tubal factor	2164 (56.86)	218 (58.76)	
Ovulatory disorder	861 (22.62)	80 (21.56)	
Diminished ovarian reserve	142 (3.73)	24 (6.47)	
Endometriosis	148 (3.89)	11 (2.96)	
Unexplained	463 (12.17)	22 (5.93)	
Others	28 (0.74)	16 (4.31)	
Ovarian stimulation protocol, n (%)			0.87
Depot GnRH agonist	1534 (40.30)	144 (38.81)	
GnRH agonist	1032 (27.12)	103 (27.76)	
GnRH antagonist	1131 (29.72)	111 (29.92)	
Micro stimulation	109 (2.86)	13 (3.50)	
No. of oocytes retrieved	10.35 ± 5.99	10.46 ± 6.18	0.73
1–5, n (%)	845 (22.20)	75 (20.22)	
6–9	1049 (27.56)	113 (30.46)	
10–15	1239 (32.55)	121 (32.61)	
≥ 16	673 (17.68)	62 (16.71)	
No. MII oocytes	9.66 ± 5.67	8.78 ± 5.40	< 0.01
No. 2PN	6.53 ± 4.25	6.25 ± 4.33	0.22
Fertilization rate (% per MII)	67.64	71.15	< 0.01
Total fertilization failure, n (%)	70 (1.84)	10 (2.70)	0.25
Cycles with no embryo viable, ^c^ n (%)	184 (4.83)	15 (4.04)	0.50
No. of embryos available	5.51 ± 3.93	5.06 ± 3.83	0.03
Blastulation rate (%)	63.79	52.70	< 0.01
No. of embryos cryopreserved	3.07 ± 2.92	2.41 ± 2.45	< 0.01

Data are expressed as mean ± SD or n (%). ^a^Including the passive smoking status; ^b^Median (Interquartile range); ^c^Including fertilization failures and arrested embryo development

*AFC *Antral follicle counts, *MII *MetaphaseII, *2PN *Two pronuclear

Despite a similar number of oocytes retrieved, the number of MIIoocytes (8.78 vs. 9.66) was significantly lower in ICSI group than in IVF group. Fertilization rate per MIIoocyte was comparable between the two groups, but blastulation rate (52.70% vs. 63.79%, *P* < 0.01) were decreased with ICSI treated cycles. There were less embryos available and cryopreserved in the ICSI group compared with the IVF group. The rate of total fertilization failure was low with similar frequency following both IVF (1.8%, 10/371) and ICSI (2.7%, 70/3086) (*P* = 0.25) (Table 1).

### Outcomes

Table 2 shows the pregnancy outcomes after the first cycles of embryo transfer. There were 199 cases with no embryos available (including fertilization failures and arrested embryo development) (15 in ICSI group and 184 in IVF group). Until the end of study period, 24 cases did not undergo embryo transfer (19 in IVF group and 5 in ICSI group). The proportion of women who had at least one transfer was 351/371 in the ICSI and 3613/3806 in the IVF group.

**Table 2:** Comparison of the first embryo transfer cycle outcomes

	IVF	ICSI	*P*-value
No. of patients	3613	351	
Cycles of embryo transfer, n (%)			
Fresh	2175 (60.20)	198 (56.41)	0.17
Frozen-thawed	1438 (39.80)	153 (43.59)	
No. of embryos transferred	1.39 ± 0.49	1.45 ± 0.50	0.03
1, n (%)	2211 (61.20)	194 (55.27)	0.08
2	1400 (38.75)	157 (44.73)	
3	2 (0.06)	0	
Embryo stage, n (%)			
Cleavage-stage	1640 (45.39)	198 (56.41)	< 0.01
Blastocyst-stage	1973 (54.61)	153 (43.59)	
Clinical pregnancy rate, n (%)	2250 (62.28)	198 (56.41)	0.03
Live birth rate, n (%)	1729 (47.85)	151 (43.02)	0.08
Plurality at birth, n (% per cycle)			
Singleton	1468 (40.63)	126 (35.90)	0.20
Twins	261 (7.22)	25 (7.12)	

Data are expressed as mean ± SD or n (%). 14 women did not undergo embryo transfer until the end of follow-up (9 in IVF group and 5 in ICSI group)

In the first transfer, the mean number of embryos transferred was 1.45 in the ICSI group versus 1.39 in the IVF group (*P* = 0.03). More cycles with a single embryo transfer were performed in the IVF group (61.2% vs 55.27%). In the ICSI group, 56.41% of the transferred embryos were in cleavage-stage, while in IVF group 54.6% of embryo transfer were in blastocyst-stage. After controlling for confounders, the pregnancy outcomes and fertilization rates did not differ between the groups (Table 3). Live birth rate after first embryo transfer was 151/351 (43.02%) in ICSI treated women and 1729/3613 (47.85%) in IVF treated women (AOR, 0.91; 95%CI, 0.72–1.15). Clinical pregnancy (56.41% vs. 62.28%, AOR, 0.89; 95% CI, 0.71–1.13) and fertilization rate per MII (71.15% vs. 67.64%, AOR, 1.10; 95% CI, 0.86–1.40) were similar between the groups. The twin birth rate per cycle after the first ET was 7.12% in the ICSI group versus 7.22% in the ICSI group (*P* = 0.20).

**Table 3:** Adjusted fertilization/clinical pregnancy/live birth rates

	OR (95%CI)		AOR (95%CI)	
	IVF	ICSI	IVF	ICSI
Fertilization^a^ (2PN/MIIoocyte)	Ref	1.18 (1.09, 1.28)	Ref	1.10 (0.86, 1.40)
Clinical pregnancy^b^	Ref	0.78 (0.63, 0.98)	Ref	0.89 (0.71, 1.13)
Live birth^b^	Ref	0.82 (0.66, 1.03)	Ref	0.91 (0.72, 1.15)

*2PN *two pronuclear, *MII *MetaphaseII, *AOR *adjusted odds ratio

^a^Model adjusted for, female age, male age, infertility duration, AFC, BMI, causing of infertility, number of oocytes retrieved and MII oocytes

^b^Model adjusted for, female age, male age, infertility duration, AFC, BMI, causing of infertility, number of oocytes retrieved, number of MIIoocytes, number and stage of embryos transferred

### Sensitive analysis

Women in the IVF group were younger than patients in ICSI group, and so it could be argued that ICSI achieved similar live birth rates despite being used in poor prognosis patients. In order to reduce the potential bias, we conducted a sensitive analysis that restricted to women aged 35 years old and over, which reduced the effect of age difference between the two groups. Patient and cycle characteristics were comparable between two groups (Additional file 2). Similar to the entire cohort, the odds of live birth, clinical pregnancy and fertilization were similar between the groups after controlling for covariates (Table 4).

**Table 4:** Adjusted fertilization/clinical pregnancy/live birth rates in women aged ≥ 35 years old^a^

	OR (95%CI)		AOR (95%CI)	
	IVF	ICSI	IVF	ICSI
Fertilization^b^ (2PN/MIIoocyte)	Ref	1.10 (0.93, 1.29)	Ref	1.06 (0.66, 1.70)
Clinical pregnancyc	Ref	0.69 (0.45, 1.04)	Ref	0.74 (0.47, 1.17)
Live birth^c^	Ref	0.59 (0.37, 0.95)	Ref	0.66 (0.39, 1.11)

*2PN *two pronuclear, *MII *MetaphaseII, *AOR *adjusted odds ratio

^a^Baseline characteristics were similar between the groups

^b^Model adjusted for, female age, male age, infertility duration, AFC, BMI, causing of infertility, number of oocytes retrieved and MIIoocytes

cModel adjusted for, female age, male age, infertility duration, AFC, BMI, causing of infertility, number of oocytes retrieved, and MIIoocytes, fertilization rate, number and stage of embryos transferred

## Discussion

This study is large data set of couples with non-male infertility in which the duration of infertility at their first IVF/ICSI treatment were analyzed. The results demonstrated that women with a duration of infertility beyond a certain turning point (4.8 years) had decreased incidence of IVF fertilization. To the best of our knowledge, this is the first study to explore the role of infertility duration on fertilization rates. Patients with long infertility duration (5 years and over) were then followed through their reproductive treatment. Our data further found that ICSI was not associated with increased likelihood of a live birth for non-male factor infertility with prolonged infertility duration. The findings further confirm the evidence comparing the efficacy of IVF and ICSI treatment and support the committee opinion of the Practice Committee of the American Society for Reproductive Medicine [12], which reviewed the apply of ICSI in non-male infertility but made no reference to prolonged infertility duration. Notably, the rate of fertilization and fertilization failure were similar between the two methods, thereby indicating that there was no advantage in using ICSI in the setting of long infertility duration.

From the clinicians’ and infertile patients’ points of view, low or failed fertilization occurred more commonly in the IVF cycles than in the ICSI cycles. In contrast to what we are inclined to think, ICSI cannot fully compensate for interval time-dependent decline of fertilization.

The risk of fertilization failure after IVF using sperm of normal quality has been reported to range from 2.1% to 10% [22–24]. Consistent with previous studies [25, 26], despite the use of ICSI, total fertilization failure occurs in 2.70% (10/371) of ICSI treated women in the present study, indicating that simple modification of fertilization methods may not settle the overall issue [27]. Failure of fertilization might be secondary to poor oocyte quality, leading to an association with poorer reproductive outcomes. According to our results, compared to cycles with embryo transfer, cycles with fertilization failure in ICSI had less MII oocytes, which has been shown to be a cause of failed fertilization [27]. Besides sperm penetration, a series of post-sperm penetration events are also essential in successful fertilization. Mature oocyte activation is indispensable for successful fertilization, which is caused by intracellular Ca^2+^ triggered by the binding of the sperm to the oolemma [28]. As the most common cause of fertilization failure after ICSI, oocyte activation deficiency is associated either with molecular sperm- or oocyte-related factors, such as phospholipase C zeta (PLCZ1) deficiency in sperm, Wee1-Like Protein Kinase 2 (WEE2) mutation in oocyte and so on [29].

Our results demonstrated lower blastulation rate with use of ICSI over conventional IVF (52.70% vs. 63.79%, *P* < 0.01). It has been reported that ICSI treatment was associated with reduced blastocyst formation [23]. In this cohort study, only women without male factor infertility were included, we assumed that the reason for low quality of injected spermatozo was unlikely. ICSI itself may be responsible to the poor embryo development. Our findings were consistent with results of a previous study showing a low implantation rate in cycles where ICSI was used, leading the authors to conclude that ICSI offered no advantage in in postfertilization reproductive outcomes over conventional IVF in cases of non–male factor infertility [30]. Moreover, as an invasive and complex technique, ICSI may cause added cost burden, oocyte damage, and potential birth defects, and developmental concerns in offspring [31]. Additional large and well-designed RCT are needed to clarify the role of ICSI in non-male factor infertility.

During the study period, of the first stimulation cycles in couples with non-male factor infertility ≥ 5 years, 3806 patients were performed with IVF (91.12%) and 371 patients (8.88%) were treated with ICSI. Based on data from National ART Service Provision Surveys [32], the proportion of ICSI in mainland China was 29.2%, which was lower than the percentage in the United States in 2019 (56.4%) [33], and much lower than that in Europe 2014 report (71.3%) [11]. The difference is probably attributable to the ICSI technique being mainly indicate for only male factor infertility or female infertility in cases of polyspermy or poor fertilization in a prior IVF cycle, as documented in ART guidelines in China.

In natural pregnancy, time of unwanted non-conception has been taken as the key factor affecting the pregnancy prospect. However, little is known about its influence of on reproductive outcomes in ART. Many women erroneously believe that ART can always address the infertility issues with no time limit [34]. On the basis of the current data, extending infertility duration decreases the incidence of fertilization and which could not be reversed by use of ICSI. Postponing the investigation in women with infertility can be taken as a source of failure and frustration [35]. Such information may help couples have well-informed perception on when to seek help and management.

This study allowed us to better understand the duration of infertility and its corresponding effects on fertilization rate. Women with longer infertility duration are likely to have an advanced age and age-related pregnancy decline has already been shown. Therefore, age and number of oocytes retrieved were taken into consideration when analyzing the association between infertility duration and reproductive outcomes. Moreover, by using the first cycle only, we were able to limit the number of ICSI cycles included in our data set due to a history of a prior cycle with poor fertilization.

There are some limitations to acknowledge. First, owing to the retrospective design of the study, we cannot exclude the presence of confounding bias, although the potential influence of variation was minimized by adjustments and multivariable regression analysis. The ICSI group had a higher age, which could mean that ICSI was performed in poorer prognosis patients. Prospective randomized studies are required to precisely compare the outcomes after IVF versus ICSI. Secondary, a post hoc power analysis was performed to determine how many cases would have been required to find a difference in live birth rate between the ICSI and IVF groups. This calculation showed that almost 9500 women should be included to show that live birth rates would have been significantly lower with ICSI than with IVF. We cannot exclude that larger number of subjects might have a statistical significance. Nevertheless, there is no evidence overall that use of ICSI improves laboratory or pregnancy outcomes in patients with long time infertility. Finally, this study was based on follow-up data from a single ART center, therefore caution is needed before generalizing the results to other populations.

## Conclusion

In conclusion, increasing awareness of the impact of infertility of on the reproductive outcomes is essential for couples to seek counseling. Use of ICSI for non-male factor infertility with 5 years and over does not demonstrate improvement in reproductive outcomes and fertilization rate. Infertile patients may benefit from an early resort to assisted reproduction treatment.

## Supplementary Information

**Additional file 1.**

**Additional file 2. **Characteristics and outcomes of patients aged 35 years and over with infertility ≥5 years.

## Data Availability

The datasets used and analyzed during the current study are available from the corresponding author on reasonable request.

## Abbreviations

ART: Assisted reproductive technology
PGT: Preimplantation genetic test
ET: Embryo transfer
AFC: Antral follicular count
BMI: Body mass index
2PN: Two pronuclear
MII: MetaphaseII
AOR: Adjusted odds ratio
CI: Confidence interval
