# Supplementary material for: Duration of infertility and assisted reproductive outcomes in non-male factor infertility: can use of ICSI turn the tide?
Source: BMC Womens Health. 2022 Nov 28;22:480. doi: 10.1186/s12905-022-02062-9 (PMC9706853; doi:10.1186/s12905-022-02062-9)
Supplement: Supplementary file 1 — Additional file 1. [file 12905_2022_2062_MOESM1_ESM.docx]

Additional file 1.

|  | Crude ß (95% CI) | *P*-value | Adjusted ß (95% CI) | *P*-value |
| --- | --- | --- | --- | --- |
| Fertilization rate per MⅡ |  |  |  |  |
| IVF |  |  |  |  |
| < 5 years | -0.06 (-0.08,0.03) | <0.01 | -0.05 (-0.08,0.03) | <0.01 |
| ≥5 years | 0.01 (-0.01,0.04) | 0.28 | -0.01 (-0.03,0.02) | 0.62 |
| ICSI | -0.04 (-0.08,0.01) | 0.11 | -0.04 (-0.09,0.01) | 0.09 |
| Fertilization rate per oocyte |  |  |  |  |
| IVF |  |  |  |  |
| < 5 years | -0.06 (-0.08,0.03) | <0.01 | -0.06 (-0.08,0.03) | <0.01 |
| ≥5 years | 0.01 (-0.01,0.04) | 0.28 | -0.01 (-0.04,0.02) | 0.57 |
| ICSI | -0.04 (-0.08,0.01) | 0.10 | -0.05 (-0.09,0.00) | 0.07 |

MⅡ, MetaphaseⅡ
