# Supplementary material for: Duration of infertility and assisted reproductive outcomes in non-male factor infertility: can use of ICSI turn the tide?
Source: BMC Womens Health. 2022 Nov 28;22:480. doi: 10.1186/s12905-022-02062-9 (PMC9706853; doi:10.1186/s12905-022-02062-9)
Supplement: Supplementary file 2 — Additional file 2. Characteristics and outcomes of patients aged 35 years and over with infertility ≥5 years. [file 12905_2022_2062_MOESM2_ESM.docx]

Additional file 2. Characteristics and outcomes of patients aged 35 years and over with infertility ≥5 years

|  | IVF | ICSI | *P*-value |
| --- | --- | --- | --- |
| Patients aged ≥ 35 years | 837 | 111 |  |
| Years of treatment, n (%) |  |  | 0.84 |
| 2017 | 219 (87.25) | 32 (12.75) |  |
| 2018 | 223 (88.84) | 28 (11.16) |  |
| 2019 | 222 (89.52) | 26 (10.48) |  |
| 2020 | 173 (87.37) | 25 (12.63) |  |
| Female age (years) | 37.23 ± 2.13 | 37.27 ± 2.21 | 0.23 |
| Male age (years) | 38.65 ± 3.99 | 39.35 ± 4.83 | 0.09 |
| Body mass index (kg/m^2^) | 23.26 ± 3.19 | 23.33 ± 2.73 | 0.82 |
| Education level, n (%) |  |  |  |
| Primary | 66 (7.89) | 9 (8.11) | 0.98 |
| High school | 528 (63.08) | 69 (62.16) |  |
| College | 243 (29.03) | 33 (29.73) |  |
| Smoker,^a^ n (%) | 171 (20.43) | 24 (21.62) | 0.77 |
| Gravidity, n (%) |  |  |  |
| 0 | 288 (34.41) | 49 (44.14) | 0.09 |
| 1 | 255 (30.47) | 25 (22.52) |  |
| ≥2 | 294 (35.13) | 37 (33.33) |  |
| No. of prior live births, n (%) |  |  |  |
| 0 | 459 (54.84) | 74 (66.67) | 0.06 |
| 1 | 333 (39.78) | 33 (29.73) |  |
| ≥2 | 45 (5.38) | 4 (3.60) |  |
| AFC, n | 9.90 ± 5.41 | 8.84 ± 4.62 | 0.05 |
| Causing of infertility, n (%) |  |  |  |
| Tubal factor | 515 (61.53) | 62 (55.86) | 0.07 |
| Ovulatory disorder | 101 (12.07) | 14 (12.61) |  |
| Diminished ovarian reserve | 69 (8.24) | 18 (16.22) |  |
| Endometriosis | 34 (4.06) | 5 (4.50) |  |
| Unexplained | 106 (12.66) | 9 (8.11) |  |
| Others | 12 (1.43) | 3 (2.70) |  |
| Ovarian stimulation protocol, n (%) |  |  |  |
| Depot GnRH agonist | 171 (20.43%) | 19 (17.12%) | 0.25 |
| GnRH agonist | 191 (22.82%) | 23 (20.72%) |  |
| GnRH antagonist | 435 (51.97%) | 59 (53.15%) |  |
| Micro stimulation | 40 (4.78%) | 10 (9.01%) |  |
| No. of oocytes retrieved | 8.26 ± 5.36 | 8.71 ± 5.92 | 0.41 |
| (%)1-5 | 298 (35.60%) | 29 (26.13%) | 0.11 |
| 6-9 | 253 (30.23%) | 45 (40.54%) |  |
| 10-15 | 198 (23.66%) | 27 (24.32%) |  |
| ≥16 | 88 (10.51%） | 10 (9.01%) |  |
| No. MⅡoocytes | 7.73 ± 5.06 | 7.12 ± 4.65 | 0.22 |
| No. 2PN | 5.35 ± 3.83 | 5.06 ± 3.87 | 0.45 |
| Fertilization rate (% per oocyte) | 64.83 | 58.12 | <0.01 |
| Fertilization rate (% per MⅡ) | 69.24 | 71.14 | 0.27 |
| Total fertilization failure, n (%) | 14 (1.67%) | 3 (2.70%) | 0.44 |
| Cycles with no embryo viable,^c^  n (%) | 42 (5.02) | 5 (4.50) | 0.82 |
| No. of embryos available | 4.54 ± 3.53 | 4.05 ± 3.27 | 0.17 |
| Blastulation rate (%) | 61.23 | 52.65 | <0.01 |
| No. of embryos cryopreserved | 2.49 ± 2.40 | 1.97 ± 2.33 | 0.03 |
| Cycles of embryo transfer | 790 | 104 |  |
| Fresh,n (%) | 361 (45.70) | 50 (48.08) | 0.65 |
| Frozen-thawed | 429 (54.30) | 54 (51.92) |  |
| No. of embryos transferred |  |  |  |
| 1, n (%) | 447 (56.58) | 65 (62.50) | 0.47 |
| 2 | 341 (43.16) | 39 (37.50) |  |
| 3 | 2 (0.25) | 0 |  |
| Embryo stage, n (%) |  |  |  |
| Cleavage-stage | 445 (56.33) | 69 (66.35) | 0.05 |
| Blastocyst-stage | 345 (43.67) | 35 (33.65) |  |
| Clinical pregnancy rate, n (%) | 408 (51.65) | 44 (42.31) | 0.07 |
| Live birth rate, n (%) | 284 (35.95) | 26 (25.00) | 0.03 |
| Plurality at birth, n (% per cycle) |  |  |  |
| Singleton | 251 (31.77) | 22 (21.15) | 0.08 |
| Twins | 33 (4.18) | 4 (3.85) |  |

Data are expressed as mean ± SD or n (%). ^a^Including the passive smoking status; ^b^Including fertilization failures and arrested embryo development.

AFC, antral follicle counts; MⅡ, MetaphaseⅡ, 2PN, two pronuclear.
